# Zone 2 Intensity: A Critical Comparison of Individual Variability in Different Submaximal Exercise Intensity Boundaries

**DOI:** 10.1155/tsm2/2008291

**Published:** 2025-02-23

**Authors:** Benedikt Meixner, Luca Filipas, Hans-Christer Holmberg, Billy Sperlich

**Affiliations:** ^1^Integrative and Experimental Exercise Science & Training, Department of Sport Science, Julius-Maximilians-Universität Würzburg, Würzburg, Germany; ^2^Department of Sport Science and Sport, Friedrich-Alexander-Universität Erlangen-Nürnberg, Erlangen, Germany; ^3^iQ-Move Praxis Fraunberger, Erlangen, Germany; ^4^Department of Biomedical Sciences for Health, Università Degli Studi di Milano, Milan, Italy; ^5^TotalEnergies Pro Cycling Team, Essarts-en-Bocage, France; ^6^Division of Machine Elements, Luleå University of Technology, Luleå, Sweden; ^7^School of Kinesiology, University of British Columbia, Vancouver, Canada

**Keywords:** aerobic exercise, exercise metabolism, moderate intensity, substrate utilization, training prescription

## Abstract

**Introduction:** Endurance athletes often utilize low-intensity training, commonly defined as Zone 2 (Z2) within a five-zone intensity model, for its potential to enhance aerobic adaptations and metabolic efficiency. This study aimed at evaluating intra- and interindividual variability of commonly used Z2 intensity markers to assess their precision in reflecting physiological responses during training.

**Methods:** Fifty cyclists (30 males and 20 females) performed both an incremental ramp and a step test in a laboratory setting, during which the power output, heart rate, blood lactate, ventilation, and substrate utilization were measured.

**Results:** Analysis revealed substantial variability in Z2 markers, with the coefficients of variation (CV) ranging from 6% to 29% across different parameters. Ventilatory Threshold 1 (VT_1_) and maximal fat oxidation (Fat_Max_) showed strong alignment, whereas fixed percentages of HR_max_ and blood lactate thresholds exhibited wide individual differences.

**Discussion:** Standardized markers for Z2, such as fixed percentages of HR_max_, offer practical simplicity but may inaccurately reflect metabolic responses, potentially affecting training outcomes. Given the considerable individual variability, particularly in markers with high CVs, personalized Z2 prescriptions based on physiological measurements such as VT_1_ and Fat_Max_ may provide a more accurate approach for aligning training intensities with metabolic demands. This variability highlights the need for individualized low-intensity training prescriptions to optimize endurance adaptations in cyclists, accommodating differences in physiological profiles and improving training specificity.

## 1. Introduction

Endurance exercise elicits a broad spectrum of metabolic and morphological adaptations in skeletal muscle (such as increased capillarization, elevated activity of enzymes involved in the mitochondrial electron transport chain, and decreased carbohydrate utilization), designed to attenuate cellular perturbations during subsequent training sessions [1]. These chronic adaptations are hypothesized to result from the cumulative effects of repeated exercise bouts, with initial signaling events triggering the adaptive processes following each session [1]. For practical application and to optimize the targeting of specific adaptations, exercise intensity is systematically categorized into distinct training zones [2], such as Zone 2, threshold [3], or SIT [4]. These zones are typically derived from various testing procedures, including ramp or incremental step testing protocols [5–8].

Depending on the sport and discipline, multiple intensity models exist, with the most commonly used frameworks being the three-zone, five-zone, and seven-zone models [2, 6, 9, 10]. These models provide a structured framework for training intensity prescription, allowing for the modulation of exercise intensity to selectively engage various energy systems (i.e., predominantly phosphagenic, glycolytic, or oxidative), muscle fiber types, and cardiovascular mechanisms [1, 11, 12]. The choice of model may vary depending on the sport and federation (e.g., Norway [13] and Germany [14]), with each framework offering a different level of granularity in intensity prescription. For instance, the three-zone model divides intensity into broad categories suitable for monitoring overall training load, while the five- and seven-zone models enable a more detailed approach, with additional zones designed to target specific physiological adaptations (e.g., neuromuscular or cardiac adaptations and upregulation of mitochondrial enzymes [15, 16]).

Although no standardized criteria currently exist for defining these training zones [6], each zone is often determined by objective physiological markers, such as heart rate and blood lactate concentration [17–28] and ventilatory response [9, 29–32], or by power output or velocity [24, 33–40]. Based on the athlete's specific needs and objectives, this zonal approach enables the individualized tailoring of training stimuli to elicit targeted metabolic and performance outcomes, such as enhancing aerobic capacity, optimizing fuel utilization [41], or improving lactate clearance efficiency [2, 42, 43].

Training in intensity Zone 2 (Z2), based on a five-zone intensity model, is particularly popular in endurance sports, especially those with decreased mechanical stress (such as cycling or cross-country skiing), due to its role in enhancing aerobic capacity, efficiency, and metabolic flexibility [44]. Z2 training is positioned in the low-intensity range, making it a foundational component of training for both elite and amateur athletes [2, 44]. The primary rationale behind extensive Z2 exercise lies in its ability to stimulate mitochondrial efficiency and enhance fat metabolism [41, 44, 45] among a variety of adaptations (e.g., insulin sensitivity, oxidative capacity, glucose control, and increased glucose stores) [46, 47]. This supports sustained energy production during long-duration efforts by preserving glycogen stores and delaying fatigue, enabling athletes to maintain high performance over extended periods—a key factor for both professional and amateur athletes [48]. While not a new concept [49], elite cyclists, including recent Tour de France champions, frequently incorporate Z2 training [50], dedicating approximately 80% of their training time to this intensity and below [51].

With the widespread availability of power meters and heart rate sensors, amateur cyclists often rely on accessible, nonlaboratory-based markers to guide their training [3]. These markers include %HR_max_, heart rate reserve (HRR), or percentages of peak power output (%PPO), which are used to target physiological goals such as maximizing fat oxidation (Fat_Max_) and staying within an “aerobic zone.” Z2 intensity typically corresponds to approximately 72%–82% of HR_max_ [10], 1.5–2.5 mmol/L blood lactate [10], or 60%–75% of functional threshold power (FTP) [3] in cycling.

Fat_Max_, in particular, has become a popular objective within Z2 training due to its role in improving endurance performance and extending time to fatigue during prolonged events [52]. To further define Z2 intensity, additional physiological indicators are employed, including Ventilatory Threshold 1 (VT_1_) [9], 65% V̇O_2peak_ [53], and blood lactate concentrations within the range of 1.5–2.5 mmol/L (BLa_1.5_, BLa_2.0_, BLa_2.5_) [10], to further help distinguish intensity boundaries.

However, despite the widespread application of Z2 training, there is growing evidence that commonly used physiological markers—such as % of HR_max_ or metabolic reference points such as capillary blood lactate thresholds (e.g., power output at 2 mmol/L blood lactate, P_2_)—may not result in consistent metabolic responses across individuals [54, 55]. This variability arises not only between individuals with different aerobic capacities but even within subgroups of athletes with similar fitness levels [55].

Such findings suggest that the conventional approach of using fixed percentages or numbers to define the upper and lower boundaries of Z2 may not ensure the desired metabolic perturbation across all athletes. Consequently, using generalized percentages or surrogate markers may result in exercise prescriptions that are either too low or too high for specific individuals, which could undermine the effectiveness of training.

To ensure a training intensity domain such as Z2 elicits the targeted metabolic load, it is essential to account for the significant interindividual variability in physiological responses. This study aimed at analyzing the extent of variability in the most commonly applied reference values for Z2 as this variability is critical for determining the precision and effectiveness of Z2 training. Specifically, we aim to determine whether the current methods for prescribing Z2 training—based on the heart rate, blood lactate, ventilatory indices, or power—consistently lead to similar exercise intensities across individuals, or if a more individualized approach is required. We hypothesized that nonphysiological Z2 demarcations based on percentages of a maximal value display a large intraindividual variation, complicating their application as Z2 training prescriptors.

By investigating this variability, we aim to provide insights into the limitations of percentage- and number-based training methods in Z2, emphasizing the need for refined approaches in prescribing low-intensity exercise to optimize endurance training for cyclists.

## 2. Methods

### 2.1. Participants

A cohort of *n* = 50 (*n* = 30 males and *n* = 20 females) experienced cyclists with more than three years of regular cycling exercise (> 2 sessions per week) were recruited for this study [56]. All participants were experienced in road cycling with clipless pedals and cycled regularly as exercise. Prior to the study, the participants were informed of the protocol and gave their written informed consent to participate. All procedures were approved by the ethical committee of Exercise Science & Training of the Faculty of Human Sciences of the University of Würzburg (EV2024/1-1004) and conducted in accordance with the Declaration of Helsinki [57, 58]. The main characteristics of the participants are summarized in Table 1.

### 2.2. Experimental Design

**Figure 1:**
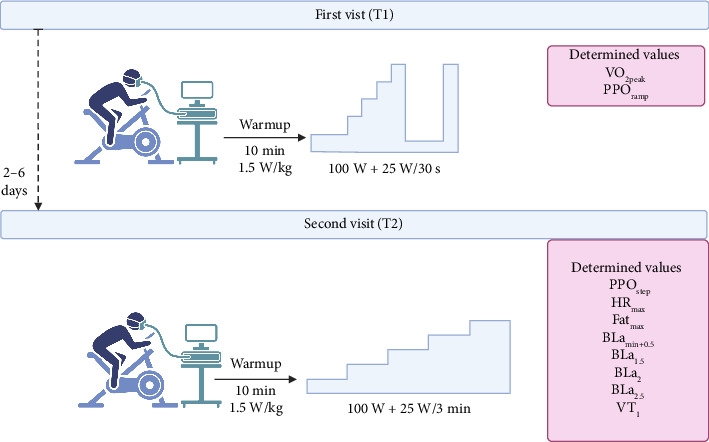
Study procedure.

Two experimental visits (T1 and T2) to the laboratory were required, which were at least 48 h apart and completed within a 7-day period to complete a ramp and a step incremental test. The overall study design is illustrated in Figure 1.

All participants were instructed to keep a nutrition diary and to repeat their usual diet for each visit within the 24 h before each experimental visit [59]. In addition, all were instructed to stay adequately hydrated, to eat a carbohydrate-rich meal (i.e., a banana and a jam sandwich) no less than 3 h before each visit, and to refrain from caffeine consumption on the day of testing. Each participant received 35 g of a carbohydrate mixture (IsoFast, DextroEnergy, Krefeld, Germany) dissolved in 500 mL of water to drink *ad libitum* during warmup and recovery periods.

### 2.3. Ramp Protocol

To determine V̇O_2peak_ and PPO_ramp_, all participants performed a ramp protocol in T1. The participants began cycling at 100 W for 2 min with a freely chosen cadence, after which the load increased by 25 W every 30 s [60]. The ramp ended when volitional exhaustion was reached, or the cadence dropped by more than 10 rpm. Following 3-min active recovery at 75 W, a verification phase at 100% of the load at the last fully completed 30 s until volitional exhaustion was performed [61]. V̇O_2peak_ was calculated as the highest value averaged over 30 s.

### 2.4. Incremental Step Protocol

To determine power output at baseline blood lactate concentration +0.5 mmol/L (BLa_min+0.5_), BLa_1.5_, BLa_2.0_, BLa_2.5_, VT_1_, 65% V̇O_2peak_, Fat_Max_, HR_72%_, and HR_82%_, a step protocol was performed (T2). The participants began the test with a freely chosen cadence but were advised to maintain their regular cadence. The test started at 100 W, with the load increasing by 25 W every 3 min. Three participants wished to start at 75 W based on previous results. The test concluded when volitional exhaustion was reached or when cadence consistently dropped by more than 10 rpm. Peak power output (PPO) during the step test was estimated using the following formula:

(1) $$\begin{array}{c} {\text{PPO}}_{\text{step}}=P_{\text{last⁣fully⁣completed⁣stage}}+\frac{t_{\text{in⁣last⁣stage}}}{180}*25. \end{array}$$

### 2.5. Cycling Ergometer

All cyclists used their own shoes and pedals for both tests, and the tests were performed on the cyclist's own personal road bike installed on a Cyclus2 ergometer (RBM, Leipzig, Germany). The Cyclus2 is an electromagnetically braked ergometer and measures power with an accuracy error of 2% according to the manufacturer. For both visits, all cyclists warmed up for 10 min cycling at 1.5 W/kg body mass, then performed a short sprint before undergoing an incremental ramp (T1) or step (T2) protocol.

### 2.6. Gas Exchange and Heart Rate Measurement

Participants were fitted with a Hans Rudolph V2 mask (Hans Rudolph, Inc, Shawnee, KS, USA), and expired gases along with breathing volume were analyzed using a Cosmed Quark CPET system (Cosmed Srl, Rome, Italy). Gas and volume analyzers were calibrated before each test using precision gas (16% O_2_ and 5% CO_2_) and a volume pump, following the manufacturer's instructions (Airgas Therapeutics, Plumsteadville, PA, USA). Gas exchange parameters were averaged over 10-s intervals, with only the final 30 s of each step being considered for analysis. VT_1_ was determined following the method described in Ref. [62] and assessed by two independent experienced researchers in exercise testing and prescription who were blinded to the identity of the athletes and the aim of the study. Power output at VT_1_ was linearly interpolated based on the test duration.

All were equipped with a Polar H10 (Polar Electro Oy, Kempele, Finland) heart rate belt. The heart rate minimum was the lowest value determined during the resting period before the warmup and thereafter. Maximum heart rate was determined as the maximal value averaged over 10 s.

### 2.7. Capillary Blood Lactate Concentration

Capillary blood samples of the left earlobe were sampled before the start of the step test and in the last 15 s of every step. The lactate concentration was measured amperometric-enzymatically employing Biosen C-Line (EKF Diagnostics, Barleben, Germany).

### 2.8. Z2 Definition and Determination of Thresholds

The following definitions were analyzed as representative benchmarks for defining “Zone 2” intensity:

• 72%–82% HR_max_ (HR_72%_, HR_82%_) and 1.5–2.5 mmol/L blood lactate used by the Norwegian Olympic Federation (BLa_1.5_, BLa_2.5_) [10].
• 65% of V̇O_2peak_ [53, 63].
• Blood lactate concentration of 2 mmol/L (BLa_2.0_) [9, 17, 64].
• Baseline blood lactate concentration + 0.5 mmol/L (BLa_min+0.5_) [65].
• First ventilatory threshold (VT_1_) [9, 66, 67].
• Maximum fat oxidation rate (Fat_Max_) [41, 45]. For the determination of Fat_Max_, standard nonprotein stoichiometric equations for moderate intensity were applied according to Jeukendrup and Wallis [68],
  (2) $$\begin{array}{c} \text{Fat oxidation}\left[\frac{g}{\min}\right]=\left(1.695*{\text{VO}}_{2}\right)-\left(1.701*{\text{VCO}}_{2}\right). \end{array}$$

### 2.9. Data Processing

All data were collected and exported in Microsoft Excel. All statistical analyses were performed in GraphPad Prism (v.10.4, Boston, MA, USA).

HR, V̇O_2_, fat oxidation, and blood lactate were matched to the corresponding power outputs at the end of each step. Slopes and intercepts of HR and V̇O_2_ were aligned with power output via individual linear regressions, based on values measured at rest, at the end of each stage, and at maximum effort. All linear regressions for HR and V̇O_2_ achieved a goodness of fit (*R*^2^ ≥ 0.95). Lactate values were interpolated using third-order polynomial regression analysis, with all regressions attaining goodness of fit (*R*^2^ ≥ 0.97).

For all blood lactate-based thresholds (BLa_1.5_, BLa_2_, BLa_2.5_, and La_min+0.5_), power at the corresponding lactate level was calculated using third-order polynomial regression, with matching HR and V̇O_2_ determined via linear regression. Nine participants did not reach a lactate value lower than 1.5 mmol/L and were excluded from this analysis. For VT_1_, power output at VT_1_ was interpolated, and matching HR and V̇O_2_ were similarly determined via linear regression. For 65% of V̇O_2max_, power output was calculated and matched to HR via linear regression. For HR_72%_ and HR_82%_, the respective percentages of HR_max_ were calculated and matched to power output and HR using linear regression. For Fat_Max_, the power output corresponding to the highest total fat oxidation in the final 30 s was determined, with matching HR and V̇O_2_ calculated via linear regression [69].

### 2.10. Statistical Analysis

All raw data were collected using Microsoft Excel (Microsoft Corp., Redmond, WA, USA). Statistical analyses (mean, standard deviations, and 95% confidence intervals) were computed with GraphPad Prism (v10.3, Boston, MA, USA). Normality of the power output, heart rate, and V̇O_2_ was assessed using the Shapiro–Wilk test, without requiring further transformation, except for power output at Fat_Max_ and corresponding V̇O_2_. Median and mean absolute deviation (MAD) are therefore given for this demarcation [70], calculated with JASP (v0.18.3). The level of significance (*α*) was set to 0.05 for all statistical analyses.

Differences between the position of markers and the respective maximum values were analyzed employing a paired mixed-effects model because of missing values in one Z2 demarcation. Tukey's multiple comparison test was applied to identify differences between the different Z2 demarcations.

The interindividual coefficient of variation (CV) was calculated as the standard deviation divided by the mean for absolute values of power, heart rate, and oxygen uptake of the given Z2 demarcations [71]. For Fat_Max_, a robust version of CV (based on robust MAD) was calculated according to Arachchige et al. [72]. Bland–Altman plots were used to assess agreement, with bias and limits of agreement calculated as the difference between Z2 demarcations and their average [73].

Differences between males and females were analyzed by independent sample Student's *t*-tests.

## 3. Results

The mean values, standard deviations, and CV for each variable reflecting Z2 in relation to the peak oxygen uptake, peak heart rate, and PPO are summarized in Table 2. The CVs ranged from 6% to 29% with HR_72%_, HR_82%_, and BLa_2.5_ displaying the lowest variation, while power output at BLa_1.5_ exhibited the highest CV.

**Figure 2:**
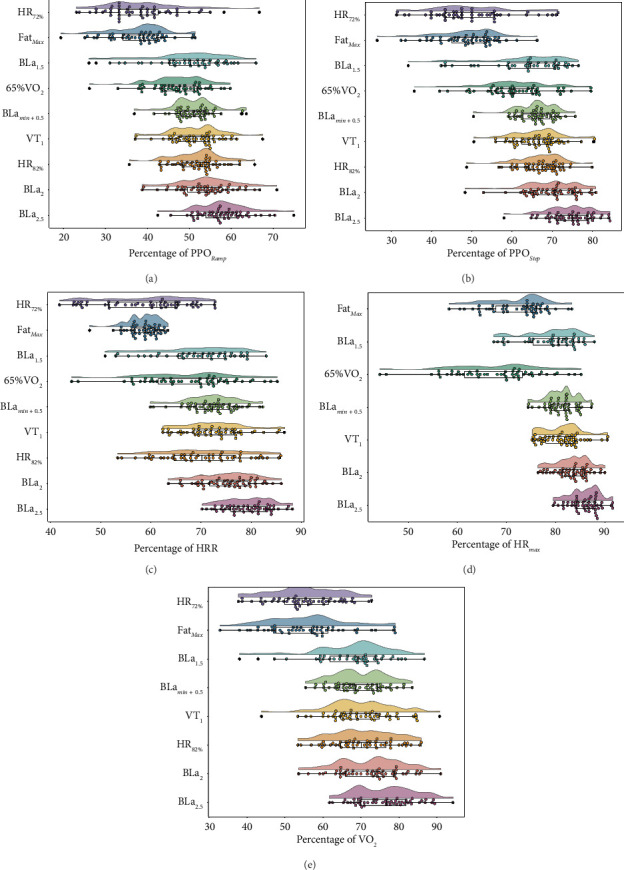
Raincloud plots illustrating Zone 2 demarcations expressed as power output, heart rate, or oxygen uptake relative to their respective maximum values.

The rain cloud plots in Figure 2 show single data points for each individual and variable related to Z2 in relation to V̇O_2peak_, HR_max_, HRR, PPO_ramp_, and PPO_step_.

**Figure 3:**
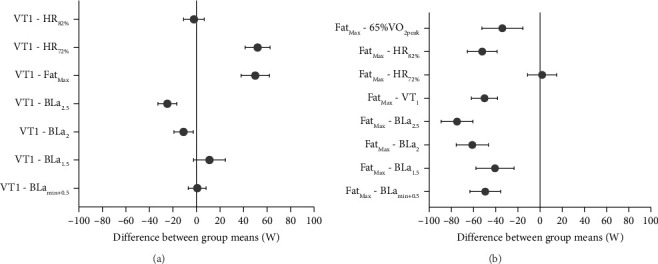
Mean difference in power output (W) between variables with statistical overlay, relative to (a) VT_1_ and (b) Fat_Max_ as reference points.

Upon analyzing the absolute power output values across different Z2 demarcations, the multiple comparison test revealed no significant differences between the combinations of BLa_min+0.5_, BLa_1.5_, VT_1_, and HR_82%_, as well as between HR_72%_ and Fat_Max_ (*p* > 0.05). However, all other pairwise comparisons demonstrated significant differences (*p* < 0.01). Figures 3(a) and 3(b) display the mean differences and 95% confidence intervals with VT_1_ and Fat_Max_ as reference markers.

When related to % of PPO_ramp_ and PPO_step_, there was similar overlay (i.e., no significant differences, *p* < 0.05) for BLa_min+0.5_ and BLa_1.5_; BLa_min+0.5_ and VT_1_; BLa_min+0.5_ and HR_82%_; BLa_1.5_ and VT_1_; BLa_1.5_ and HR_82%_; BLa_1.5_ and 65% V̇O_2peak_; BLa_2_ and HR_82%_; HR_72%_ and Fat_Max_; and VT_1_ and HR_82%_. In contrast, all other comparisons displayed significant differences (*p* < 0.01).

When expressed as % of peak oxygen uptake, the mean values of BLa_min+0.5_ and BLa_1.5_; BLa_min+0.5_ and VT_1_; BLa_min+0.5_ and HR_82%_; BLa_1.5_ and VT_1_; BLa_1.5_ and HR_82%_; BLa_2_ and HR_82%_; HR_72%_ and Fat_Max_; and VT_1_ and HR_82%_ did not differ (*p* > 0.05). In contrast, all other combinations showed significant differences (*p* > 0.01).

When related to % HR_max_, no differences (*p* > 0.01) were found for BLa_min+0.5_ and BLa_1.5_; BLa_min+0.5_ and BLa_2_; BLa_min+0.5_ and VT_1_; BLa_1.5_ and VT_1_; BLa_1.5_ and 65% V̇O_2peak_; BLa_2_ and BLa_2.5_; and BLa_2_ and VT_1_.

**Figure 4:**
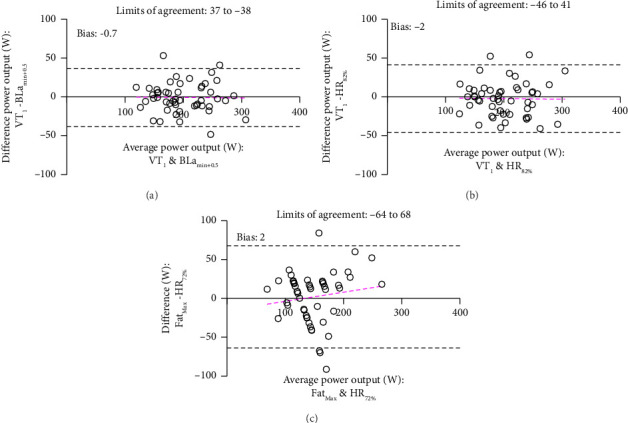
Bland–Altman plots of differences in power output between variables with statistical overlay: (a) VT_1_ vs. BLa_min+0.5_, (b) VT_1_ vs. HR_82%_, and (c) Fat_Max_ vs. HR_72%_.

To illustrate the interconnection between physiologically determined Z2 demarcations and standardized ones, Figure 4 illustrates bias and limits of agreement of VT_1_ and BLa_min+0.5_; VT_1_ and HR_82%_; and Fat_Max_ and HR_72%_.

Differences between males and females were found for power output at BLa_min+0.5_ (*p* = 0.0023), HR_72%_ (*p* = 0.0197), HR_82%_ (*p* = 0.0105), BLa_2_ (*p* = 0.0116), and BLa_2.5_ (*p* = 0.0167) in relation to PPO_step_ and for heart rate at Fat_Max_ in relation to HR_max_ (*p* = 0.0188). Females displayed a higher ratio of their heart rate at Fat_Max_ compared to the maximal value than males. In all ratios of the power output, females displayed a lower ratio to PPO_step_.

## 4. Discussion

The primary finding of this study is the significant interindividual and methodological variability observed in variables defining Z2 boundaries, including the heart rate, capillary blood lactate concentration, oxygen uptake, and power output. All these variables, commonly employed to characterize Z2, displayed notable individual differences. Based on our hypothesis, we thereby infer that Z2 demarcations not based on physiological measures are potentially suitable as proxy measures for Z2. However, the large intraindividual variability leads us to discourage from their general application in place of physiologically based diagnostic procedures.

From the mean statistical analysis, BLa_min+0.5_, VT_1_, and HR_82%_, as well as Fat_Max_ and HR_72%_, yielded comparable values. BLa_1.5_ was also comparable to BLa_min+0.5_, VT_1_, and HR_82%_; however, only 41 of the 50 participants achieved a power output corresponding to this demarcation.

The present study reveals substantial variability in the heart rate and power output thresholds associated with Z2, underscoring the challenges in establishing standardized training prescriptions. Specifically, Z2 thresholds for the heart rate and power output exhibited CV exceeding 20%, which emphasizes the extent of interindividual differences in physiological responses, even among individuals with comparable aerobic capacities. These findings are consistent with prior research indicating heterogeneous metabolic responses at similar percentage-based thresholds (e.g., %V̇O_2peak_ and FTP%), highlighting the limitations of universal Z2 guidelines for precision in low-intensity endurance training [54, 55]. Given this inherent variability in defining Z2, focusing on variables with relatively low CV may provide a more consistent basis for comparison across individuals or groups, thereby enhancing the validity of Z2-based training prescriptions.

This variability likely originates from several individual physiological factors that influence energy production and utilization during exercise. The key aspects include the following:

i. Mitochondrial density [74], which determines the cell's capacity for oxidative metabolism and affects aerobic performance [1].
ii. Lactate kinetics, which dictates the rate at which lactate is produced, utilized, and cleared from the bloodstream.
iii. Oxygen transport efficiency, encompassing the cardiovascular and respiratory systems' ability to deliver oxygen to active muscles [75].
iv. Muscle fiber composition, particularly the ratio of slow-twitch to fast-twitch fibers, which impacts the preferred metabolic pathways during exercise [76, 77].

Together, these elements shape how blood lactate is produced, distributed, and cleared in response to various exercise intensities [78]. Since these factors vary significantly between individuals, they play a central role in determining the physiological response at submaximal intensities, thus impacting the accuracy and utility of training zones when prescribed based on fixed percentage guidelines [55]. As a result, it is unlikely that all athletes will achieve optimal adaptations from Z2 training prescribed solely by a fixed percentage of heart rate (e.g., 72%–82% of HR_max_), as some may overestimate their intended intensity, while others may underestimate it [79, 80]. Although heart rate-based measures generally exhibit less interindividual variation, a seemingly minor difference of 5% can translate to approximately 10 bpm, potentially posing a substantial difference in training intensity for the athlete.

Amateur cyclists often rely on accessible, nonlaboratory-based markers such as %HR_max_, HRR, or %PPO as references to target physiological goals such as Fat_Max_ or other metrics referring to an “aerobic zone,” defined, e.g., by low blood lactate concentrations [3]. However, Figures 2(a), 2(b), 2(c), 2(d), and 2(e) illustrate that using such simplified intensity prescriptions can lead to highly heterogeneous physiological responses. In fact, we observed significant interindividual variability in Z2 intensity markers, such as HR_72%_–HR_82%_, a widely used definition within the five-zone model [10]. Acknowledging this variability, relying on fixed percentages of HR_max_ or blood lactate concentration for prescribing Z2 training may not yield similar anticipated results for all athletes.

Research indicates that males and females exhibit distinct physiological characteristics, particularly in their ratios of lactate threshold 1 (LT1) to PPO [81]. Our analysis also displayed differences between males and females in some cases. However, the differences we found were the opposite of those mentioned by Benítez-Muñoz [81]. This is possibly caused by methodological differences in both studies. Nonetheless, these findings underscore that there is a discrepancy between males and females and employing standardized percentages may not be equally suitable across the two groups.

The differences between males and females can be attributed to factors such as muscle composition, hormonal influences, and metabolic capacities [81]. For instance, women typically have a higher proportion of type I muscle fibers and greater capillary density, which enhance oxidative metabolism and endurance performance [82]. Hormonal variations, including differences in estrogen and progesterone levels, also play a role in substrate utilization and muscle function [82]. When training zones are determined using a standardized percentage of PPO, these inherent sex differences mean that the prescribed training intensities may not align equitably for both groups. Consequently, males and females could experience different metabolic responses to the same training stimulus, impacting the effectiveness of their training regimens. This discrepancy underscores the need for personalized training programs that consider individual physiological profiles. This ensures that each athlete receives the appropriate metabolic stimuli necessary to maximize performance and achieve training goals.

VT_1_ marks the transition from predominantly aerobic metabolism to increased anaerobic contribution, aligning with a shift from fat to carbohydrate as the primary fuel source [41, 83]. In this case, using VT_1_ to determine the upper boundary of the low-intensity exercise domain, including Z2, compared to relying on HR_72%_–HR_82%_ may represent a more individualized approach to ensure athletes train within an intensity range that optimally stimulates fat oxidation and promotes glycogen sparing [55, 84] while also facilitating intensity control [13].

The present data indicate that power at VT_1_ and Fat_Max_ may overlap in some cyclists; however, Table 2 shows that Fat_Max_ is generally ∼25% lower than VT_1_—indicating that these markers represent distinct intensity domains [85, 86]. Fat_Max_ is often the rationale for many athletes to exercise at this intensity due to its association with optimal fat oxidation [45], which is particularly relevant for enhancing metabolic flexibility, i.e., the ability to transition between fats and carbohydrates as fuel sources depending on the availability and metabolic demand [41], improving endurance, and supporting body composition goals [87, 88]. For endurance athletes, training at Fat_Max_ can contribute to improved energy efficiency, allowing for the conservation of glycogen stores during prolonged exercise [45]. Additionally, exercising at this intensity may appeal to recreational athletes focused on weight management, as it targets fat as a major fuel source [87, 88].

Therefore, if Fat_Max_ is the desired physiological metric, we recommend directly assessing Fat_Max_ to optimize fat oxidation during exercise, rather than relying on surrogate intensity markers. It is also noteworthy that while training at Fat_Max_ may not elicit superior adaptations compared to other intensities [89], it provides an intensity with a high workload-to-carbohydrate utilization ratio, which is advantageous for those aiming to maximize fat utilization while minimizing carbohydrate consumption during exercise [90].

Blood lactate concentration also offers insights into energy metabolism during exercise [91]. The “P_2_” threshold, which indicates the onset of rapid lactate accumulation, suggests a transition to greater carbohydrate utilization and reduced fat oxidation [41, 91]. However, our findings indicate that using P_2_ as a proxy for, e.g., Fat_Max_, is not advisable, as in many instances, P_1.5_ more accurately reflects the threshold below which lipid metabolism is maximized [45]. Moreover, relying on fixed blood lactate concentrations lacks the individualized precision recommended for performance enhancement. This is particularly relevant for blood lactate assessments, where adopting an individualized approach involves simple recalculations rather than changes to the underlying methodology.

### 4.1. The Dilemma of Individualized vs. Universal Approaches to Z2 Training Prescription

The findings of this study highlight the challenges in defining and prescribing Z2 training intensities using standard methods. The significant interindividual variability observed in Z2 markers underscores the limitations of generalized indicators, such as fixed percentages of heart rate maximum (%HR_max_), PPO, or standardized blood lactate concentrations. Traditional benchmarks such as 72%–82% HR_max_ or blood lactate levels between 1.5 and 2.5 mmol/L [92] offer a broad framework, but they do not consistently reflect the true metabolic demands across athletes, explaining heterogeneous training adaptations.

Our results indicate that relying on universal benchmarks can result in training loads that are either insufficiently challenging or overly taxing, which can diminish training effectiveness [13]. This variation poses a fundamental dilemma for exercise physiologists and coaches: should training prescriptions prioritize the simplicity of generalized markers, or the precision of individualized markers that better capture the athlete's unique physiological responses?

Furthermore, we are not yet certain which Z2 boundary metric is optimal for achieving specific training outcomes, as this depends on the targeted training goals. The ideal boundary metric may vary based on factors such as the athlete's training background, specific sports demands, and desired adaptations (e.g., enhancing fat oxidation, maximizing mitochondrial density, or improving metabolic flexibility) [44, 93–95]. Generalized prescriptions based on %HR_max_ or fixed blood lactate concentrations are popular due to their ease of use, but our data suggest that they do not adequately account for interindividual variability, potentially leading to mismatches between prescribed and actual Z2 boundaries, affecting the desired physiological load.

Adopting an individualized exercise intensity approach is essential to improve training precision and effectiveness [96–99]. Specifically, Z2 intensity can be more accurately determined through individualized physiological markers such as VT1, BLa_min+0.5_, or Fat_Max_. Fat_Max_, for instance, allows for precise targeting of intensities that optimize fat oxidation [45], an adaptation crucial for endurance performance [41]. This metric is particularly valuable for athletes aiming to enhance endurance, support glycogen sparing, or improve body composition.

However, each marker comes with trade-offs, and the choice of metric should consider athlete-specific factors such as their metabolic efficiency, current fitness level, competitive goals, and access to specialized equipment [100, 101]. VT_1_ or markers with low CV may provide more accurate and consistent Z2 training intensities across athletes, allowing coaches to better target desired adaptations such as improved mitochondrial function or enhanced fat metabolism [44]. Although generalized markers are convenient and provide useful guidelines where direct measurements are not feasible, individualized markers—by aligning training more closely with each athlete's unique physiological profile—can optimize training outcomes more effectively [102].

In summary, individualized prescriptions for Z2 training represent a significant advancement in tailoring low-intensity training for endurance adaptation. While generalized approaches are practical, individualized markers such as VT_1_ or Fat_Max_ provide a more precise framework for achieving targeted metabolic adaptations, creating a balanced approach to enhancing training efficacy and athlete performance across diverse training goals [102].

### 4.2. Training Intensity Distributions

A similar dilemma applies not only to the prescription of training through defined zones but also to the description of training intensity distributions (TIDs) [103]. Meta-analyses of TIDs across endurance disciplines reveal a wide range of definitions for low-intensity Z2 training [2, 92]. Our data suggest that these differing definitions lead to substantial variation in actual training intensity and metabolic load, which in turn affects the comparability across studies, methodologies, and disciplines [103].

Our findings reveal that the relatively small limits of agreement and minimal bias between VT_1_ and BLa_min+0.5_ (Figure 4(a)) suggest that these two individually determined threshold markers are well-aligned and thus comparable for defining Z2. While other Z2 demarcations exhibit some degree of overlap, the Bland–Altman analysis highlights increased bias and wider limits of agreement in the comparison of VT_1_ to HR_82%_. Importantly, VT_1_ and BLa_min+0.5_ represent demarcations based on physiological markers, whereas HR_82%_ coincides with these thresholds without being grounded in a direct metabolic response to incremental exercise [104]. In the case of Fat_Max_ and HR_72%_, although no mean differences were detected, the large limits of agreement observed indicate that these two markers are not interchangeable as Z2 demarcations due to their considerable variability.

For other threshold demarcations, however, intraindividual differences become more pronounced, resulting in distinct metabolic challenges depending on the chosen threshold definition. This raises a critical question about the “true” nature of Z2: although often equated with low-intensity exercise, Z2 can represent quite different physiological demands depending on the variable used to define it [6]. This variability suggests that Z2 may not be a single, universally applicable intensity domain, but rather a concept that encompasses different metabolic states and adaptations depending on the specific marker [6].

### 4.3. Strengths, Limitations, and Future Directions

A notable strength of this study is the use of a cohort of experienced amateur cyclists, providing insight into the variability of physiological markers even among trained individuals. The inclusion of both ramp and incremental step testing protocols also allowed for a comprehensive evaluation of multiple Z2 intensity markers.

However, several limitations should be acknowledged. The cross-sectional nature of this study prevented us from evaluating the long-term effects of individualized versus generalized Z2 on training. Additionally, the method used to calculate Fat_Max_ may have introduced variability that influenced the precision of this marker. We consider it a strength of our study that both a ramp protocol and a step protocol were performed. However, when comparing metrics across both protocols, this bears the potential to skew the analysis. Furthermore, our study included only an analysis of cycling and was unable to consider differences in modality [105].

Future studies should include a broader range of endurance athletes as well as modalities and consider additional metrics such as functional threshold power (FTP) and muscle oxygen saturation or employ longitudinal designs to assess the impact of individualized prescriptions over time. Additionally, the possible differences in metabolic response to a single dose of constant Z2 training defined by various demarcations (e.g., Fat_Max_, VT_1_, BLa_2_) may provide further insight into this area.

## 5. Perspective

This study underscores the complexity and challenge of defining Z2 training in a manner that aligns with individual physiological responses, highlighting the limitations of one-size-fits-all prescriptions. Acknowledging the significant interindividual variability across markers such as heart rate, lactate thresholds, oxygen uptake, and power output, there is a compelling case for moving toward more personalized training frameworks. While generalized markers offer convenience and can serve as useful starting points, our findings emphasize that tailored prescriptions based on individualized markers—such as VT1, BLa_min+0.5_, or Fat_Max_—may provide a more accurate reflection of each athlete's unique metabolic profile, thereby enhancing the potential for targeted adaptations. Moving forward, the integration of personalized Z2 markers into both research and practical training programs could refine endurance training, enabling athletes to optimize their low-intensity efforts and ultimately their performance outcomes. This perspective encourages further investigation into the nuances of individualized versus standardized Z2 prescriptions, potentially leading to more refined guidelines for practitioners in sports science and coaching.

## 6. Conclusion

The present data underscore the substantial interindividual variability in physiological responses to Z2 intensity prescribed using fixed markers such as % HR_max_ or blood lactate concentrations, challenging the efficacy of traditional methods and highlighting the need for personalized training approaches. By adopting individualized markers such as VT_1_ and Fat_Max_, athletes can achieve more tailored intensity prescriptions that optimize fat metabolism and aerobic performance. Notably, Z2—often equated with low-intensity exercise—can represent distinct metabolic demands depending on the boundaries used, suggesting that it may not be a single, universally applicable intensity domain. Instead, Z2 may encompass varied metabolic states depending on the threshold marker, reinforcing the value of individualized approaches for effective endurance training.

## Figures and Tables

**Table 1:** Anthropometric data of participants.

Variable	All (*n* = 50)	Male (*n* = 30)	Female (*n* = 20)
Age (years)	31.2 ± 7.8	34.3 ± 7.8	26.5 ± 5.0
Height (cm)	177.5 ± 9.3	182.6 ± 7.0	169.8 ± 6.6
Body mass (kg)	71.7 ± 12.4	78.6 ± 10.3	61.4 ± 6.6
Body fat (%)	13.9 ± 4.8	11.7 ± 3.8	17.1 ± 4.2
Relative oxygen uptake (mL/min/kg)	54.1 ± 8.1	56.0 ± 8.6	51.2 ± 6.6

**Table 2:** Oxygen uptake, heart rate, and power output as mean ± SD, % of maximal respective value, and coefficient of interindividual variation for different Zone 2 demarcations.

	Oxygen uptake (mL/min) at	% peak oxygen uptake	CV%	HR (b/min)	% of HR_max_	CV%	Power output (W)	% of PPO_ramp_	CV%
BLa_min+_0.5	2677 ± 576	69.4 ± 6.7	21.5	146 ± 10	81.2 ± 3.0	7.0	199 ± 45	51.2 ± 4.8	22.8
BLa_1.5_	2654 ± 680	65.1 ± 13.2	25.6	141 ± 10	78.1 ± 6.9	7.6	194 ± 56	46.0 ± 13.6	29.0
BLa_2.0_	2803 ± 661	72.5 ± 8.3	23.6	150 ± 9	83.4 ± 3.3	6.2	211 ± 53	54.1 ± 6.7	24.9
BLa_2.5_	2951 ± 661	76.4 ± 7.7	22.4	154 ± 9	86.0 ± 3.0	6.0	224 ± 52	57.8 ± 6.1	23.3
VT_1_	2690 ± 612	69.8 ± 8.9	22.7	146 ± 11	81.4 ±0 .3.9	7.3	200 ± 45	51.5 ± 5.9	22.8
65% V̇O_2peak_	2513 ± 546	—	21.7	140 ± 15	78.2 ± 6.0	10.8	183 ± 49	47.1 ± 6.5	26.0
Fat_Max_	2155 ± 407	56.0 ± 10.7	18.8	130 ± 12	72.3 ± 5.7	9.5	125 ± 37	38.7 ± 8.9	29.6
HR_72%_	2128 ± 525	55.2 ± 8.8	24.7	—	—	—	148 ± 41	38.0 ± 6.7	27.6
HR_82%_	2710 ± 592	70.3 ± 8.2	21.9	—	—	—	202 ± 46	51.9 ± 5.6	22.7
Maximum value	3866 ± 840	—	21.7	179 ± 10		5.7	387 ± 73 (Ramp)	—	18.9
		—					300 ± 56 (Step)	—	18.7

## Data Availability

The data that support the findings of this study are available on reasonable request from the corresponding author. The data are not publicly available due to privacy or ethical restrictions.

## Nomenclature

BLa: Capillary blood lactate
CI: Confidence intervals
CV: Coefficient of variation
Fat_Max_: Intensity corresponding to peak fat oxidation
FTP: Functional threshold power
HR: Heart rate
HR_max_: Maximal heart rate
HRR: Heart rate reserve
MAD: Median absolute deviation
PPO_ramp_: Peak power output during the ramp test
PPO_step_: Peak power output during the step test
T1: First visit to the laboratory
T2: Second visit to the laboratory
V̇O_2peak_: Peak oxygen uptake
VT_1_: Ventilatory threshold 1
Z2: Zone 2

## References

[1] Hawley J. A. (2002). Adaptations of Skeletal Muscle to Prolonged, Intense Endurance Training. *Clinical and Experimental Pharmacology and Physiology*.

[2] Sperlich B., Matzka M., Holmberg H. C. (2023). The Proportional Distribution of Training by Elite Endurance Athletes at Different Intensities During Different Phases of the Season. *Frontiers in Sports and Active Living*.

[3] Allen H., Coggan A. R., McGregor S. (2019). Training and Racing With a Power Meter. *VeloPress*.

[4] Sloth M., Sloth D., Overgaard K., Dalgas U. (2013). Effects of Sprint Interval Training on VO2max and Aerobic Exercise Performance: A Systematic Review and Meta-Analysis. *Scandinavian Journal of Medicine & Science in Sports*.

[5] Faude O., Kindermann W., Meyer T. (2009). Lactate Threshold Concepts: How Valid Are They?. *Sports Medicine*.

[6] Jamnick N. A., Pettitt R. W., Granata C., Pyne D. B., Bishop D. J. (2020). An Examination and Critique of Current Methods to Determine Exercise Intensity. *Sports Medicine*.

[7] Sandbakk Ø, Holmberg H. C. (2017). Physiological Capacity and Training Routines of Elite Cross-Country Skiers: Approaching the Upper Limits of Human Endurance. *International Journal of Sports Physiology and Performance*.

[8] Jones A. M., Doust J. H. (1998). Assessment of the Lactate and Ventilatory Thresholds by Breathing Frequency in Runners. *Journal of Sports Sciences*.

[9] Seiler K. S., Kjerland G. Ø (2006). Quantifying Training Intensity Distribution in Elite Endurance Athletes: Is There Evidence for an “Optimal” Distribution?. *Scandinavian Journal of Medicine & Science in Sports*.

[10] Sylta Ø, Tønnessen E., Seiler S. (2014). From Heart-Rate Data to Training Quantification: A Comparison of 3 Methods of Training-Intensity Analysis. *International Journal of Sports Physiology and Performance*.

[11] Zierath J. R., Hawley J. A. (2004). Skeletal Muscle Fiber Type: Influence on Contractile and Metabolic Properties. *PLoS Biology*.

[12] Laursen P. B. (2010). Training for Intense Exercise Performance: High-Intensity or High-Volume Training?. *Scandinavian Journal of Medicine & Science in Sports*.

[13] Seiler S. (2010). What Is Best Practice for Training Intensity and Duration Distribution in Endurance Athletes?. *International Journal of Sports Physiology and Performance*.

[14] Weichenberger M., Esefeld K., Müller S. (2023). Kardiopulmonale Leistungsdiagnostik Beim Spitzensportler. *Herzschrittmachertherapie & Elektrophysiologie*.

[15] Gibala M. J., Bostad W., McCarthy D. G. (2019). Physiological Adaptations to Interval Training to Promote Endurance. *Current Opinion in Physiology*.

[16] Benítez-Flores S., Castro F. A. d. S., Lusa Cadore E., Astorino T. A. (2023). Sprint Interval Training Attenuates Neuromuscular Function and Vagal Reactivity Compared With High-Intensity Functional Training in Real-World Circumstances. *The Journal of Strength & Conditioning Research*.

[17] Arne G., Stephen S., Eike E. (2009). Training Methods and Intensity Distribution of Young World-Class Rowers. *International Journal of Sports Physiology and Performance*.

[18] Schmitt L., Bouthiaux S., Millet G. P. (2021). Eleven Years’ Monitoring of the World’s Most Successful Male Biathlete of the Last Decade. *International Journal of Sports Physiology and Performance*.

[19] Schumacher Y. O., Mueller P. (2002). The 4000-m Team Pursuit Cycling World Record: Theoretical and Practical Aspects. *Medicine & Science in Sports & Exercise*.

[20] Solli G. S., Tønnessen E., Sandbakk Ø (2017). The Training Characteristics of the World’s Most Successful Female Cross-Country Skier. *Frontiers in Physiology*.

[21] Steinacker J. M., Lormes W., Kellmann M. (2000). Training of Junior Rowers Before World Championships. Effects on Performance, Mood State and Selected Hormonal and Metabolic Responses. *The Journal of Sports Medicine and Physical Fitness*.

[22] Tjelta L. I. (2013). A Longitudinal Case Study of the Training of the 2012 European 1500 M Track Champion. *International Journal of Applied Sports Sciences*.

[23] Tønnessen E., Sylta Ø, Haugen T. A., Hem E., Svendsen I. S., Seiler S. (2014). The Road to Gold: Training and Peaking Characteristics in the Year Prior to a Gold Medal Endurance Performance. *PLoS One*.

[24] Rothschild J. A., Delcourt M., Maunder E., Plews D. J. (2021). Racing and Training Physiology of an Elite Ultra-Endurance Cyclist: Case Study of 2 Record-Setting Performances. *International Journal of Sports Physiology and Performance*.

[25] Matzka M., Leppich R., Holmberg H. C., Sperlich B., Zinner C. (2021). The Relationship Between the Distribution of Training Intensity and Performance of Kayak and Canoe Sprinters: A Retrospective Observational Analysis of One Season of Competition. *Frontiers in Sports and Active Living*.

[26] Bellinger P., Arnold B., Minahan C. (2020). Quantifying the Training-Intensity Distribution in Middle-Distance Runners: The Influence of Different Methods of Training-Intensity Quantification. *International Journal of Sports Physiology and Performance*.

[27] Mujika I., Chatard J. C., Busso T., Geyssant A., Barale F., Lacoste L. (1995). Effects of Training on Performance in Competitive Swimming. *Canadian Journal of Applied Physiology*.

[28] Heck H., Wackerhage H. (2024). The Origin of the Maximal Lactate Steady State (MLSS). *BMC Sports Science, Medicine and Rehabilitation*.

[29] Lucía A., Hoyos J., Pardo J., Chicharro J. L. (2000). Metabolic and Neuromuscular Adaptations to Endurance Training in Professional Cyclists: A Longitudinal Study. *The Japanese Journal of Physiology*.

[30] Muñoz I., Seiler S., Bautista J., España J., Larumbe E., Esteve-Lanao J. (2014). Does Polarized Training Improve Performance in Recreational Runners?. *International Journal of Sports Physiology and Performance*.

[31] Seiler S., Jøranson K., Olesen B. V., Hetlelid K. J. (2013). Adaptations to Aerobic Interval Training: Interactive Effects of Exercise Intensity and Total Work Duration. *Scandinavian Journal of Medicine & Science in Sports*.

[32] Lucía A., Hoyos J., Santalla A., Pérez M., Chicharro J. L. (2002). Kinetics of VO(2) in Professional Cyclists. *Medicine & Science in Sports & Exercise*.

[33] Billat V., Lepretre P. M., Heugas A. M., Laurence M. H., Salim D., Koralsztein J. P. (2003). Training and Bioenergetic Characteristics in Elite Male and Female Kenyan Runners. *Medicine & Science in Sports & Exercise*.

[34] Billat V. L., Demarle A., Slawinski J., Paiva M., Koralsztein J. P. (2001). Physical and Training Characteristics of Top-Class Marathon Runners. *Medicine & Science in Sports & Exercise*.

[35] Kenneally M., Casado A., Gomez-Ezeiza J., Santos-Concejero J. (2022). Training Characteristics of a World Championship 5000-m Finalist and Multiple Continental Record Holder Over the Year Leading to a World Championship Final. *International Journal of Sports Physiology and Performance*.

[36] Kenneally M., Casado A., Gomez-Ezeiza J., Santos-Concejero J. (2021). Training Intensity Distribution Analysis by Race Pace vs. Physiological Approach in World-Class Middle- and Long-Distance Runners. *European Journal of Sport Science*.

[37] Leo P., Spragg J., Simon D., Lawley J. S., Mujika I. (2020). Training Characteristics and Power Profile of Professional U23 Cyclists throughout a Competitive Season. *Sports*.

[38] van Erp T., Sanders D., de Koning J. J. (2020). Training Characteristics of Male and Female Professional Road Cyclists: A 4-Year Retrospective Analysis. *International Journal of Sports Physiology and Performance*.

[39] Matzka M., Leppich R., Sperlich B., Zinner C. (2022). Retrospective Analysis of Training Intensity Distribution Based on Race Pace Versus Physiological Benchmarks in Highly Trained Sprint Kayakers. *Sports Medicine-Open*.

[40] Spragg J., Leo P., Swart J. (2023). The Relationship Between Training Characteristics and Durability in Professional Cyclists Across a Competitive Season. *European Journal of Sport Science*.

[41] San-Millan I., Brooks G. A. (2018). Assessment of Metabolic Flexibility by Means of Measuring Blood Lactate, Fat, and Carbohydrate Oxidation Responses to Exercise in Professional Endurance Athletes and Less-Fit Individuals. *Sports Medicine*.

[42] Emhoff C.-A. W., Messonnier L. A. (2023). Concepts of Lactate Metabolic Clearance Rate and Lactate Clamp for Metabolic Inquiry: A Mini-Review. *Nutrients*.

[43] Donovan C. M., Brooks G. A. (1983). Endurance Training Affects Lactate Clearance, Not Lactate Production. *American Journal of Physiology-Endocrinology And Metabolism*.

[44] San-Millán I. (2023). The Key Role of Mitochondrial Function in Health and Disease. *Antioxidants*.

[45] Jeukendrup A., Achten J. (2001). Fatmax: A New Concept to Optimize Fat Oxidation During Exercise?. *European Journal of Sport Science*.

[46] Thyfault J. P., Bergouignan A. (2020). Exercise and Metabolic Health: Beyond Skeletal Muscle. *Diabetologia*.

[47] Jones A. M., Carter H. (2000). The Effect of Endurance Training on Parameters of Aerobic Fitness. *Sports Medicine*.

[48] Seiler S. (2024). It’s About the Long Game, Not Epic Workouts: Unpacking HIIT for Endurance Athletes. *Applied Physiology Nutrition and Metabolism*.

[49] Joyner M. J. (2025). Training Elite Athletes: 50 Years of Thinking about Practice and Research for Endurance Sports. *Scandinavian Journal of Medicine & Science in Sports*.

[50] Attia P. (2024). The Meteoric Rise of Tadej Pogačar: From Prodigy to Cycling Legend. *Drive*.

[51] Sanders D., Myers T., Akubat I. (2017). Training-Intensity Distribution in Road Cyclists: Objective Versus Subjective Measures. *International Journal of Sports Physiology and Performance*.

[52] Klaris M., Cubel C., Bruun T. (2024). Performance and Fatigue Patterns in Elite Cyclists During 6 H of Simulated Road Racing. *Scandinavian Journal of Medicine & Science in Sports*.

[53] Gant N., Williams C., King J., Hodge B. J. (2004). Thermoregulatory Responses to Exercise: Relative Versus Absolute Intensity. *Journal of Sports Sciences*.

[54] Benítez-Muñoz J. A., Benito P. J., Guisado-Cuadrado I., Cupeiro R., Peinado A. B. (2024). Differences in the Ventilatory Thresholds in Treadmill According to Training Status in 971 Males and 301 Females: A Cross-Sectional Study. *European Journal of Applied Physiology*.

[55] Scharhag-Rosenberger F., Meyer T., Gässler N., Faude O., Kindermann W. (2010). Exercise at Given Percentages of VO2max: Heterogeneous Metabolic Responses Between Individuals. *Journal of Science and Medicine in Sport*.

[56] Meixner B. J., Nusser V., Koehler K., Sablain M., Boone J., Sperlich B. (2024). Relationship of Peak Capillary Blood Lactate Accumulation and Body Composition in Determining the Mechanical Energy Equivalent of Lactate During Sprint Cycling. *European Journal of Applied Physiology*.

[57] Harriss D. J., Atkinson G. (2009). International Journal of Sports Medicine-Ethical Standards in Sport and Exercise Science Research. *International Journal of Sports Medicine*.

[58] World Medical Association (2013). World Medical Association Declaration of Helsinki: Ethical Principles for Medical Research Involving Human Subjects. *JAMA*.

[59] Jeacocke N. A., Burke L. M. (2010). Methods to Standardize Dietary Intake Before Performance Testing. *International Journal of Sport Nutrition and Exercise Metabolism*.

[60] Adam J., Ohmichen M., Ohmichen E. (2015). Reliability of the Calculated Maximal Lactate Steady State in Amateur Cyclists. *Biology of Sport*.

[61] Midgley A. W., Carroll S. (2009). Emergence of the Verification Phase Procedure for Confirming ‘True’ V̇O_2max_. *Scandinavian Journal of Medicine & Science in Sports*.

[62] Keir D. A., Iannetta D., Mattioni Maturana F., Kowalchuk J. M., Murias J. M. (2022). Identification of Non-Invasive Exercise Thresholds: Methods, Strategies, and an Online App. *Sports Medicine*.

[63] McLellan T. M., Skinner J. S. (1981). The Use of the Aerobic Threshold as a Basis for Training. *Canadian Journal of Applied Sport Sciences*.

[64] Hartmann U., Mader A., Hollmann W. (1990). Heart Rate and Lactate During Endurance Training Programs in Rowing and Its Relation to the Duration of Exercise by Top Elite Rowers. *FISA Coach*.

[65] Beaver W. L., Wasserman K., Whipp B. J. (1986). A New Method for Detecting Anaerobic Threshold by Gas Exchange. *Journal of Applied Physiology*.

[66] Esteve-Lanao J., Juan A. F. S., Earnest C. P., Foster C., Lucia A. (2005). How Do Endurance Runners Actually Train? Relationship With Competition Performance. *Medicine & Science in Sports & Exercise*.

[67] Zapico A., Calderon F., Benito P. (2007). Evolution of Physiological and Haematological Parameters With Training Load in Elite Male Road Cyclists: A Longitudinal Study. *The Journal of Sports Medicine and Physical Fitness*.

[68] Jeukendrup A. E., Wallis G. A. (2005). Measurement of Substrate Oxidation During Exercise by Means of Gas Exchange Measurements. *International Journal of Sports Medicine*.

[69] Lounana J., Campion F., Noakes T. D., Medelli J. (2007). Relationship Between %HRmax, %HR Reserve, %V̇O2max, and %V̇O2 Reserve in Elite Cyclists. *Medicine & Science in Sports & Exercise*.

[70] Davies L., Gather U., Gentle J. E., Härdle W. K., Mori Y. (2012). Robust Statistics. *Handbook of Computational Statistics: Concepts and Methods*.

[71] Lovie P. (2005). Coefficient of Variation. *Encyclopedia of Statistics in Behavioral Science*.

[72] Arachchige C., Prendergast L. A., Staudte R. G. (2022). Robust Analogs to the Coefficient of Variation. *Journal of Applied Statistics*.

[73] Martin Bland J., Altman D. G. (1986). Statistical Methods for Assessing Agreement Between Two Methods of Clinical Measurement. *The Lancet*.

[74] Barclay C. J. (2017). Energy Demand and Supply in Human Skeletal Muscle. *Journal of Muscle Research & Cell Motility*.

[75] Mortensen S. P., Damsgaard R., Dawson E. A., Secher N. H., González-Alonso J. (2008). Restrictions in Systemic and Locomotor Skeletal Muscle Perfusion, Oxygen Supply and VO2 During High-Intensity Whole-Body Exercise in Humans. *The Journal of Physiology*.

[76] Hargreaves M., Spriet L. L. (2020). Skeletal Muscle Energy Metabolism During Exercise. *Nature Metabolism*.

[77] Smith J. A. B., Murach K. A., Dyar K. A., Zierath J. R. (2023). Exercise Metabolism and Adaptation in Skeletal Muscle. *Nature Reviews Molecular Cell Biology*.

[78] Brooks G. A. (2007). Lactate: Link Between Glycolytic and Oxidative Metabolism. *Sports Medicine*.

[79] MacInnis M. J., Gibala M. J. (2017). Physiological Adaptations to Interval Training and the Role of Exercise Intensity. *The Journal of Physiology*.

[80] Coffey V. G., Hawley J. A. (2007). The Molecular Bases of Training Adaptation. *Sports Medicine*.

[81] Benítez-Muñoz J. A., Rojo-Tirado M. Á, Benito Peinado P. J., Murias J. M., González-Lamuño D., Cupeiro R. (2025). Greater Relative First and Second Lactate Thresholds in Females Compared With Males: Consideration for Exercise Prescription. *International Journal of Sports Physiology and Performance*.

[82] Esbjörnsson-Liljedahl M., Bodin K., Jansson E. (2002). Smaller Muscle ATP Reduction in Women Than in Men by Repeated Bouts of Sprint Exercise. *Journal of Applied Physiology*.

[83] Michallet A. S., Tonini J., Regnier J. (2008). Methodological Aspects of Crossover and Maximum Fat-Oxidation Rate Point Determination. *Diabetes and Metabolism*.

[84] Benitez-Muñoz J. A., Guisado-Cuadrado I., Rojo-Tirado M. A. (2025). Females Have Better Metabolic Flexibility in Different Metabolically Challenging Stimuli. *Applied Physiology Nutrition and Metabolism*.

[85] Bircher S., Knechtle B. (2004). Relationship Between Fat Oxidation and Lactate Threshold in Athletes and Obese Women and Men. *Journal of Sports Science and Medicine*.

[86] Ferri Marini C., Tadger P., Chávez-Guevara I. A. (2022). Factors Determining the Agreement Between Aerobic Threshold and Point of Maximal Fat Oxidation: Follow-Up on a Systematic Review and Meta-Analysis on Association. *International Journal of Environmental Research and Public Health*.

[87] Jiang Y., Tan S., Wang Z., Guo Z., Li Q., Wang J. (2020). Aerobic Exercise Training at Maximal Fat Oxidation Intensity Improves Body Composition, Glycemic Control, and Physical Capacity in Older People With Type 2 Diabetes. *Journal of Exercise Science & Fitness*.

[88] Tan S., Wang J., Cao L., Guo Z., Wang Y. (2016). Positive Effect of Exercise Training at Maximal Fat Oxidation Intensity on Body Composition and Lipid Metabolism in Overweight Middle-Aged Women. *Clinical Physiology and Functional Imaging*.

[89] Schwindling S., Scharhag-Rosenberger F., Kindermann W., Meyer T. (2014). Limited Benefit of Fatmax-Test to Derive Training Prescriptions. *International Journal of Sports Medicine*.

[90] Beneke R. Experiment and Computer-Aided Simulation: Complementary Tools to Understand Exercise Metabolism. *Biochemical Society Transactions*.

[91] Achten J., Jeukendrup A. E. (2004). Relation Between Plasma Lactate Concentration and Fat Oxidation Rates Over a Wide Range of Exercise Intensities. *International Journal of Sports Medicine*.

[92] Stöggl T. L., Sperlich B. (2015). The Training Intensity Distribution Among Well-Trained and Elite Endurance Athletes. *Frontiers in Physiology*.

[93] Achten J., Venables M. C., Jeukendrup A. E. (2003). Fat Oxidation Rates Are Higher During Running Compared With Cycling Over a Wide Range of Intensities. *Metabolism*.

[94] Capostagno B., Bosch A. (2010). Higher Fat Oxidation in Running Than Cycling at the Same Exercise Intensities. *International Journal of Sport Nutrition and Exercise Metabolism*.

[95] Goodpaster B. H., Sparks L. M. (2017). Metabolic Flexibility in Health and Disease. *Cell Metabolism*.

[96] Lee D., Son J.-Y., Ju H.-M., Won J.-H., Park S.-B., Yang W.-H. (2021). Effects of Individualized Low-Intensity Exercise and Its Duration on Recovery Ability in Adults. *Healthcare*.

[97] Gronwald T., Törpel A., Herold F., Budde H. (2020). Perspective of Dose and Response for Individualized Physical Exercise and Training Prescription. *Journal of Functional Morphology and Kinesiology*.

[98] Sperlich B., Gronwald T. (2024). Madness or Progress? the Dilemma of Standardizing Exercise Physiology Thresholds. *The Journal of Physiology*.

[99] Tschakert G., Handl T., Weiner L. (2022). Exercise Duration: Independent Effects on Acute Physiologic Responses and the Need for an Individualized Prescription. *Physiological Reports*.

[100] Stisen A. B., Stougaard O., Langfort J., Helge J. W., Sahlin K., Madsen K. (2006). Maximal Fat Oxidation Rates in Endurance Trained and Untrained Women. *European Journal of Applied Physiology*.

[101] Coggan A. R., Raguso C. A., Gastaldelli A., Sidossis L. S., Yeckel C. W. (2000). Fat Metabolism During High-Intensity Exercise in Endurance-Trained and Untrained Men. *Metabolism*.

[102] Wackerhage H., Schoenfeld B. J. (2021). Personalized, Evidence-Informed Training Plans and Exercise Prescriptions for Performance, Fitness and Health. *Sports Medicine*.

[103] Burnley M., Bearden S. E., Jones A. M. (2022). Polarized Training Is Not Optimal for Endurance Athletes. *Medicine & Science in Sports & Exercise*.

[104] Mann T., Lamberts R. P., Lambert M. I. (2013). Methods of Prescribing Relative Exercise Intensity: Physiological and Practical Considerations. *Sports Medicine*.

[105] Knechtle B., Müller G., Willmann F., Kotteck K., Eser P., Knecht H. (2004). Fat Oxidation in Men and Women Endurance Athletes in Running and Cycling. *International Journal of Sports Medicine*.

